# Supplementary material for: The Probiotic Kluyveromyces lactis JSA 18 Alleviates Obesity and Hyperlipidemia in High-Fat Diet C57BL/6J Mice
Source: Foods. 2024 Apr 7;13(7):1124. doi: 10.3390/foods13071124 (PMC11011337; doi:10.3390/foods13071124)
Supplement: Supplementary file 1 [file foods-13-01124-s001.zip › foods-2918917-supplementary.pdf]

Figure S1

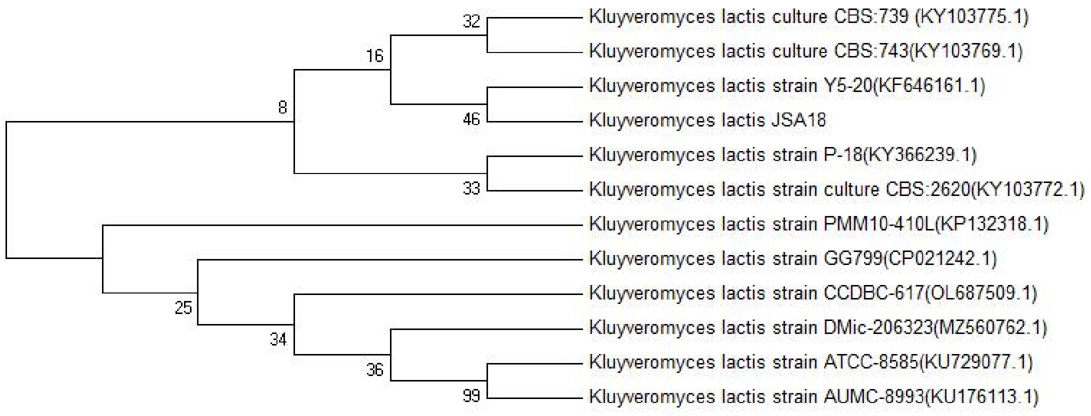

Figure S1. Phylogenetic evolutionary tree of Kluyveromyces lactis JSA18

Figure S2

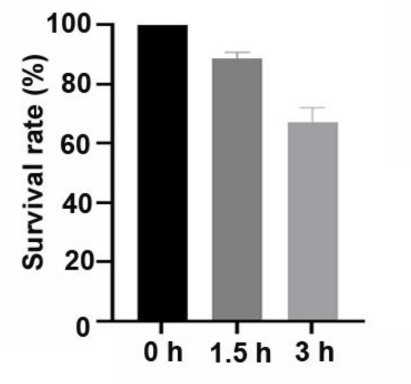

Figure S2. The survival rate of K. lactate after adding simulated gastric fluid (1.5 h) and synthetic duodenal fluid (3 h).

Figure S3

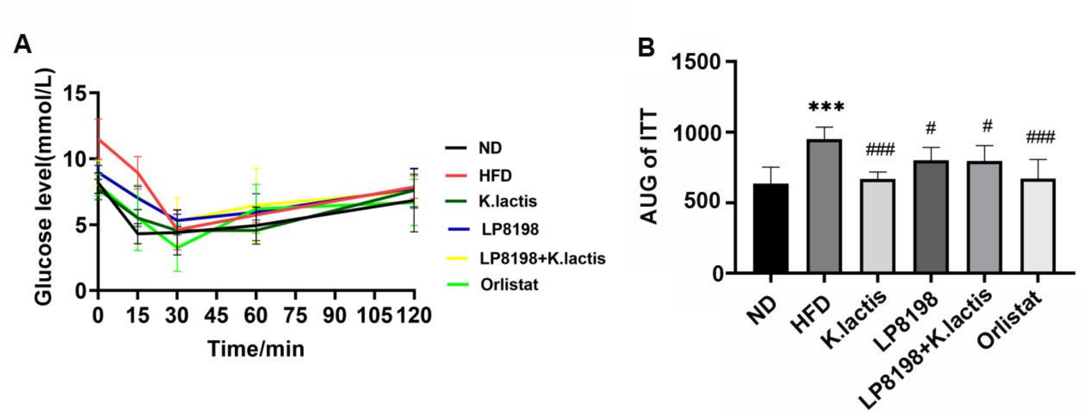

Figure S3. The effect of probiotic strains on insulin resistance in the mice fed a high-fat diet. (A) Insulin resistance was detected in each group of mice. (B) The area under the curve of insulin resistance was calculated in each group of mice. Statistical significance was evaluated using one-way ANOVA followed by a Dunnett's post hoc test. ***P< 0.005 compared to the ND group; $^{\#}$P< 0.05 and $^{\#\#\#}$ P < 0.005 compared to the HFD group.

Figure S4

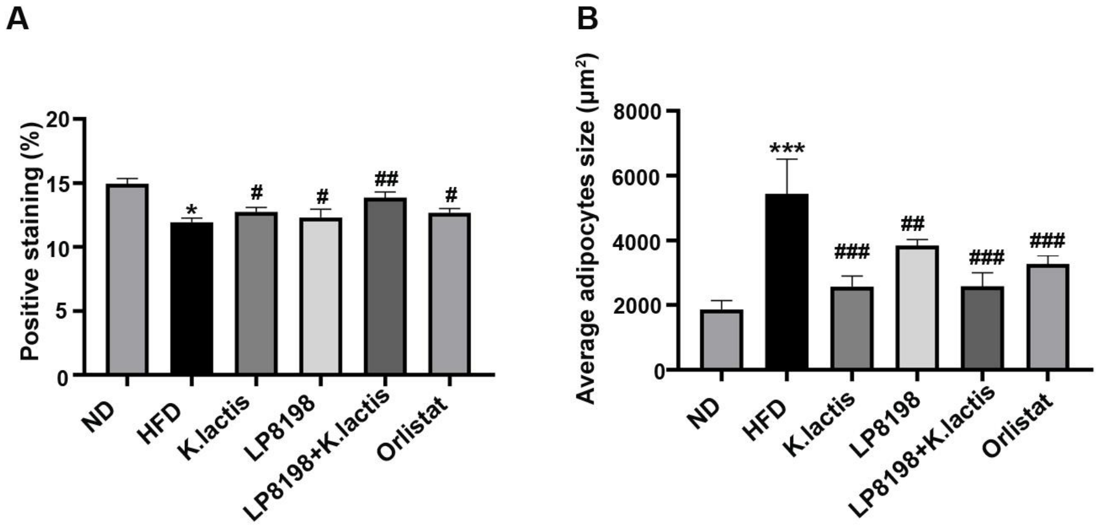

Figure S4. The quantification of liver pathologies for Figure 1J (A) and adipocyte morphology for Figure 1I (B).Statistical significance was evaluated using one-way ANOVA followed by a Dunnett's post hoc test. *P< 0.05 and ***P< 0.005 compared to the ND group; $^{\#}$P< 0.05, $^{\#\#}$P< 0.01 and $^{\#\#\#}$ P < 0.005 compared to the HFD group.

Table S1

Table S1. The self-polymerization rate, hydrophobic rate and cell adhesion rate of *K.lactis.*

Time	Self-polymerization rate (%)	Hydrophobicity rate (%)	Adhesion rate (%)
1 h	33.7	52.3	-
2 h	45.3	71.2	-
4 h	87.7	80.2	44.5
